# Spectrum of Non-Obstructive Coronary Artery Disease and Its Relationship with Atrial Fibrillation

**DOI:** 10.3390/jcm13164921

**Published:** 2024-08-21

**Authors:** Alexandru-Florinel Oancea, Paula Cristina Morariu, Ana Maria Buburuz, Ionela-Larisa Miftode, Radu Stefan Miftode, Ovidiu Mitu, Alexandru Jigoranu, Diana-Elena Floria, Amalia Timpau, Andrei Vata, Claudia Plesca, Gina Botnariu, Alexandru Burlacu, Dragos-Viorel Scripcariu, Mitea Raluca, Magdalena Cuciureanu, Daniela Maria Tanase, Irina Iuliana Costache-Enache, Mariana Floria

**Affiliations:** 1 Department of Internal Medicine I, Faculty of Medicine, “Grigore T. Popa” University of Medicine and Pharmacy, 700115 Iasi, Romania; alexandru.oancea@umfiasi.ro (A.-F.O.); radu-stefan.miftode@umfiasi.ro (R.S.M.); ovidiu.mitu@umfiasi.ro (O.M.); jigoranu.alexandru@yahoo.ro (A.J.); diana-elena.iov@d.umfiasi.ro (D.-E.F.); amalia.timpau@umfiasi.ro (A.T.); alexandru.burlacu@umfiasi.ro (A.B.); dragos-viorel.scripcariu@umfiasi.ro (D.-V.S.); daniela.tanase@umfiasi.ro (D.M.T.); irina.costache@umfiasi.ro (I.I.C.-E.); floria.mariana@umfiasi.ro (M.F.); 2 Saint Spiridon Emergency Hospital, 700115 Iasi, Romania; eosofina.botnariu@umfiasi.ro; 3 Department of Internal Medicine II, Faculty of Medicine, “Grigore T. Popa” University of Medicine and Pharmacy, 700115 Iasi, Romania; ionela-larisa.miftode@umfiasi.ro (I.-L.M.); andrei.vata@umfiasi.ro (A.V.); claudia-elena-g-plesca@umfiasi.ro (C.P.); 4 St Parascheva Clinical Hospital of Infectious Diseases, 700116 Iasi, Romania; 5 Unit of Diabetes, Nutrition and Metabolic Diseases, “Grigore T. Popa” University of Medicine and Pharmacy, 700115 Iasi, Romania; 6 Cardiovascular Disease Institute, 700503 Iasi, Romania; 7 Regional Institute of Oncology, 700483 Iasi, Romania; 8 Faculty of Medicine Victor Papilian, University of Lucian Blaga, 550169 Sibiu, Romania; daria.mitea@ulbsibiu.ro; 9 Department of Pharmacology, “Grigore T. Popa” University of Medicine and Pharmacy, 700115 Iasi, Romania; mag.cuciureanu@umfiasi.ro

**Keywords:** coronary ischemia, non-obstructive coronary artery disease, INOCA, MINOCA, ANOCA, atrial fibrillation, begets

## Abstract

This article aims to analyze the relationship between non-obstructive coronary artery disease (NOCAD) and atrial fibrillation (AF), exploring the underlying pathophysiological mechanisms and implications for clinical management. NOCAD and AF are prevalent cardiovascular conditions that often coexist, yet their interrelation is not well understood. NOCAD can lead to ischemic necrosis of cardiomyocytes and their replacement with fibrous tissue, sustaining focal ectopic activity in atrial myocardium. Atrial fibrillation, on the other hand, the most common sustained cardiac arrhythmia, is able to accelerate atherosclerosis and increase oxygen consumption in the myocardium, creating a mismatch between supply and demand, and thus promoting the development or worsening of coronary ischemia. Therefore, NOCAD and AF seem to be a complex interplay with one begets another.

## 1. Introduction

Non-obstructive coronary artery disease (NOCAD) and atrial fibrillation (AF) are two prevalent cardiovascular conditions that pose significant challenges in clinical practice due to their complex interplay and shared risk factors. Non-obstructive coronary artery disease, characterized by coronary artery stenosis of less than 50%, has emerged as a distinct entity with important implications for cardiovascular health. On the other hand, AF, the most frequent arrhythmia, is associated with an increased risk of stroke, heart failure, and mortality. The coexistence of NOCAD and AF presents a unique clinical scenario that warrants further investigation to optimize patient management and outcomes [1].

The prevalence of NOCAD has been steadily increasing, particularly in patients with symptoms suggestive of myocardial ischemia but without significant coronary artery stenosis on angiography. This subset of patients often presents with chest pain, dyspnea, and other anginal symptoms, posing a diagnostic challenge for clinicians. Recent advances in imaging modalities, such as coronary computed tomography angiography (CCTA) and invasive physiologic assessments, have improved the ability to identify and characterize NOCAD, shedding light on its pathophysiology and clinical significance [2].

Atrial fibrillation, characterized by rapid and irregular atrial electrical activity, is a common arrhythmia affecting millions of individuals worldwide. It is associated with an increased risk of thromboembolic events, including stroke, as well as heart failure and cardiovascular mortality. The presence of AF in patients with NOCAD further complicates the management of these individuals, as it may influence treatment decisions and outcomes [3].

There is a strong dual relationship between NOCAD and AF: the first one may promote through ischemia the development of focal ectopic activity in the left atrium, sustaining in this way AF. On the other hand, AF is able to generate an inflammatory response by mechanisms not yet fully understood, which will lead to the acceleration of atherosclerosis, and, moreover, by increasing oxygen consumption, it may generate a mismatch between demands and blood supply in the myocardium, generating angina in patients with NOCAD [4,5].

Understanding the complex relationship between NOCAD and AF is essential for optimizing patient care and improving clinical outcomes. This article aims to explore the dual relationship and the implications of the coexistence of NOCAD and AF. By elucidating the mechanisms underlying these conditions and their interplay, more effective strategies may be developed for risk stratification, treatment, and follow-up in this unique patient population.

## 2. Non-Obstructive Coronary Artery Disease: ANOCA, INOCA, and MINOCA

Coronary artery disease (CAD) is traditionally associated with significant obstructive lesions in the coronary arteries. However, many patients present with symptoms of myocardial ischemia or even myocardial infarction (MI) without significant coronary artery obstruction. This has led to the classification of NOCAD, which is a clinical condition characterized by the presence of coronary atherosclerosis without significant luminal obstruction, typically defined as less than 50% stenosis on CCTA or invasive angiogram. Unlike obstructive CAD, NOCAD presents a unique diagnostic and therapeutic challenge due to its subtler manifestation and complex pathophysiology [6,7].

NOCAD is increasingly recognized in clinical practice, particularly due to the widespread use of advanced imaging techniques. It is more prevalent among women than men and is often observed in patients with risk factors such as hypertension, diabetes, and hyperlipidemia. Studies indicate that NOCAD may account for up to 20–30% of patients presenting with chest pain and evidence of ischemia but without significant obstructive lesions on coronary angiography [8].

The pathophysiology of NOCAD involves several mechanisms, including endothelial dysfunction, microvascular dysfunction, and diffuse atherosclerotic changes. Endothelial dysfunction impairs the ability of the coronary arteries to dilate, leading to reduced blood flow and myocardial ischemia. Microvascular dysfunction involves the small coronary vessels, which may contribute to ischemic symptoms despite the absence of large vessel obstruction. Additionally, diffuse atherosclerotic changes may cause a gradual reduction in coronary flow reserve, exacerbating ischemic conditions [9,10].

Patients with NOCAD often present with symptoms similar to those with obstructive CAD, including chest pain (angina), dyspnea, and fatigue. However, the symptoms may be more variable and less predictable. Importantly, NOCAD can lead to significant morbidity, including recurrent hospitalizations and reduced quality of life. It is also associated with an increased risk of adverse cardiovascular events, such as MI and heart failure [8,11].

The diagnosis of NOCAD typically involves a combination of clinical assessment, non-invasive imaging, and invasive coronary angiography. Key diagnostic tools include:

- Coronary Angiography: this remains the gold standard for visualizing coronary anatomy. In NOCAD, angiography reveals less than 50% stenosis in the coronary arteries [12].
- Intravascular Ultrasound (IVUS) and Optical Coherence Tomography (OCT): these modalities provide detailed images of the coronary vessel wall, helping to identify atherosclerotic plaques and assess their composition [13,14].
- Coronary Flow Reserve (CFR), Fractional Flow Reserve (FFR), and Instantaneous Wave-Free Ratio (IFR): these functional assessments measure the physiological impact of coronary lesions [15,16].
- Non-Invasive Imaging: techniques such as stress echocardiography, cardiac MRI, positron emission tomography, and CCTA may identify ischemia and assess coronary anatomy without the need for invasive procedures [17].

**Figure 1:**
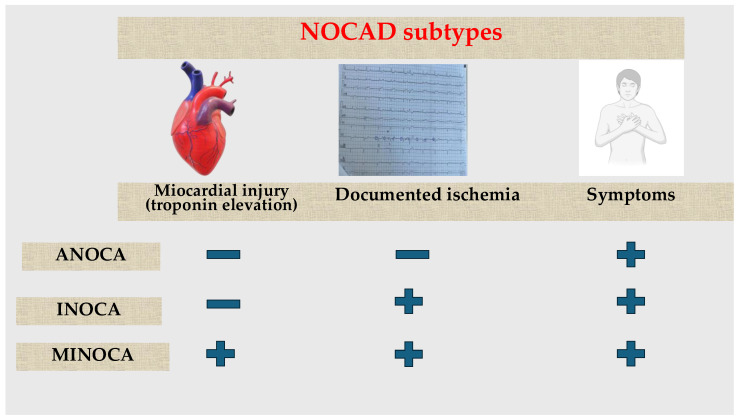
The differences between NOCAD subtypes: ANOCA, INOCA, and MINOCA. ANOCA: Angina with Non-Obstructive Coronary Arteries; INOCA: Ischemia with Non-Obstructive Coronary Arteries; MINOCA: Myocardial Infarction with Non-Obstructive Coronary Arteries.

There are more subtypes of NOCAD depending on clinical presentation, documented ischemia, and the presence of myocardial injury, defined by elevated myocardial cytolysis enzymes: Angina with Non-Obstructive Coronary Arteries (ANOCA), Ischemia with Non-Obstructive Coronary Arteries (INOCA), and Myocardial Infarction with Non-Obstructive Coronary Arteries (MINOCA) (Figure 1) [18].

Both ANOCA and INOCA refer to patients who experience angina (chest pain or dyspnea—these symptoms are often exertional and relieved by rest or nitrates, mirroring the presentation of obstructive CAD) but have non-obstructive coronary arteries on angiography and without signs of myocardial injury such as troponin elevation. The difference between them is that the first one presents without electrocardiogram (ECG) changes, stress echocardiography, or cardiac MRI suggesting ischemia; meanwhile, in the second one, non-invasive tests will show signs of ischemia [19,20].

The pathophysiological mechanisms of ANOCA and INOCA include:

- Microvascular dysfunction: impaired regulation of blood flow in the small coronary vessels, leading to insufficient oxygen supply to the myocardium caused by structural remodeling of the microvasculature, which will lead to fixed reduced microcirculatory conductance or vasomotor disorders affecting the coronary arterioles, which will cause dynamic arteriolar obstruction (the mechanism for microvascular angina) [21].
- Vasospasm: transient constriction of the epicardial coronary arteries, leading to reduced blood flow and ischemia (the mechanism for epicardial vasospastic angina) [22].

The diagnosis involves non-invasive tests to detect ischemia (which will differentiate ANOCA from INOCA) and confirm the absence of obstructive coronary arteries via angiography (Table 1).

FFR and IFR are used to differentiate borderline stenoses and classify them as obstructive CAD or NOCAD. FFR is the ratio of mean distal coronary pressure to mean aortic pressure at maximal hyperemia and an abnormal FFR is defined as ≤0.80. IFR is a non-hyperemic pressure ratio during diastole, an abnormal value being ≤0.89 [16].

Diagnostic options for coronary function testing include inducing steady-state hyperemia (by using adenosine to achieve endothelium-independent vasodilation) and the calculation of coronary flow reserve (through thermodilution using a pressure–temperature sensor guidewire in the left anterior descending artery or through Doppler flow velocity). Most studies show the prognostic value of thermodilution-based CFR has used a cut-off value of 2 [27,28].

To evaluate the microvascular resistance, two indexes may be calculated using the methods presented before the index of microvascular resistance (IMR), which may be calculated as the product of distal coronary pressure at maximal hyperemia multiplied by the hyperemic mean transit time (a value ≥ 25 is representative of microvascular dysfunction) and the hyperemic myocardial velocity resistance (HMR) index, a Doppler-based index, which may be calculated by dividing intracoronary pressure by hyperemic flow velocity (a value > 1.9 was an independent predictor of recurrent chest pain) [29,30,31].

MINOCA patients, on the other hand, present with signs and symptoms consistent with an acute myocardial infarction. This includes chest pain at rest, often with accompanying symptoms such as sweating and nausea. Importantly, these patients exhibit elevated cardiac biomarkers and may show ischemic changes on an ECG [32].

MINOCA encompasses a broader range of pathophysiological mechanisms, including:

- plaque disruption: rupture or erosion of atherosclerotic plaques that do not cause significant luminal obstruction but can lead to thrombus formation and downstream microembolization [33,34].
- spontaneous coronary artery dissection (SCAD). In general, it is present in young women, who may also present fibromuscular dysplasia as a cause. There are four types of SCAD based on angiography appearance (Table 2) [35,36].
- coronary artery spasm: severe transient constriction of a coronary artery, potentially leading to myocardial infarction [33].
- microvascular dysfunction: similar to INOCA, impaired function of the microvasculature may contribute to ischemia and infarction [37].
- coronary embolism or thrombosis: embolic events or thrombus formation in non-obstructive coronary arteries [38].

MINOCA Type 2 refers to myocardial infarction resulting from a supply–demand mismatch rather than a primary coronary event mentioned before. One notable cause of this mismatch is high-rate atrial fibrillation, which can precipitate MINOCA Type 2 by significantly increasing myocardial oxygen demand while simultaneously impairing coronary perfusion. The rapid and irregular heart rates characteristic of AF may lead to increased myocardial oxygen demand (by increasing heart rate), reduced diastolic filling time (high atrial rates reduce the time available for ventricular filling during diastole, which is critical for coronary perfusion), and coronary microvascular dysfunction (the irregular and rapid heart rates can lead to endothelial dysfunction and microvascular impairment, further compromising myocardial perfusion) [39,40].

**Figure 2:**
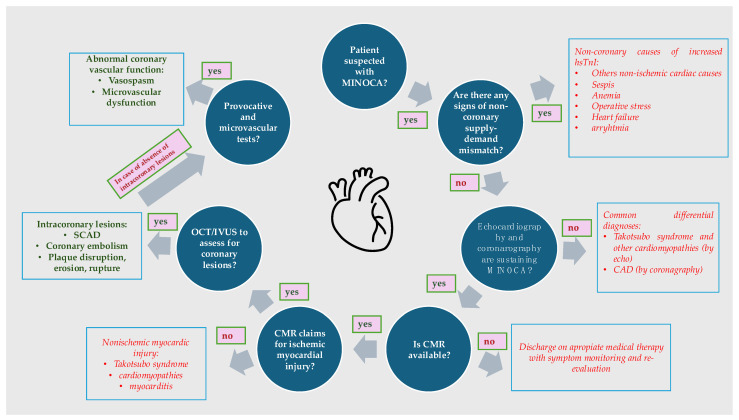
Diagnostic tools in patients suspected of MINOCA. CAD: coronary artery disease; CMR: cardiac magnetic resonance; IVUS: intravascular ultrasound; OCT: optical coherence tomography; SCAD: spontaneous coronary artery dissection.

The diagnosis starts with fulfilling the criteria for myocardial infarction (elevated cardiac biomarkers, symptoms, and ECG changes) and confirming non-obstructive coronary arteries on angiography. Additional diagnostic steps may include cardiac MRI (to identify myocardial scarring, inflammation, or other structural abnormalities), intravascular imaging (techniques like IVUS and OCT to detect subtle plaque disruptions or thrombi), and laboratory tests (to evaluate potential causes like thrombophilia or autoimmune disorders) (Figure 2) [32,38].

MINOCA must be differentiated from takotsubo cardiomyopathy (also known as stress-induced cardiomyopathy, which is characterized by transient left ventricular dysfunction due to a transient increased release of catecholamines) and from myocarditis (inflammation of the myocardium due to infectious or autoimmune causes that may mimic MI) [41,42,43].

Management of NOCAD is tailored to the underlying cause and includes antiplatelet therapy (aspirin and/or other antiplatelet agents such as P2Y12 inhibitors, which may be used in patients with plaque disruption to prevent thrombotic events), statins (these agents help stabilize atherosclerotic plaques and reduce the risk of cardiovascular events), beta-blockers, ACE inhibitors (which may improve endothelial function and reduce cardiovascular risk in patients with hypertension or diabetes), and specific interventions (such as calcium channel blockers and nitrates for coronary artery spasm). Moreover, lifestyle modifications should be carried out, such as adopting a heart-healthy diet, eliminating tobacco use to reduce endothelial damage and atherosclerosis progression, weight and stress management, and regular physical activity should be promoted to improve cardiovascular fitness and endothelial function [44,45,46].

ANOCA and INOCA treatment primarily focuses on alleviating symptoms and improving quality of life, using antianginal medications such as beta-blockers and nitrates. For patients with microvascular angina, drugs like ranolazine and trimetazidine may also be beneficial and, in cases of vasospastic angina, calcium channel blockers and nitrates are preferred. MINOCA treatment, on the other hand, starts with standard acute myocardial infarction protocols, including dual antiplatelet therapy, statins, ACE inhibitors, ARBs, and beta-blockers, the most important being the identification and treatment of the underlying cause. Not least for all of the NOCAD subtypes, the control of risk factors is very important (hypertension, diabetes, obesity, dyslipidemia). Patients with MINOCA especially benefit from aggressive LDL cholesterol control. Statins are the first-line therapy, aiming for LDL levels below 55 mg/dL. PCSK9 inhibitors or ezetimibe may be added if target LDL levels are not achieved with statins alone [47,48,49].

## 3. Coronary Ischemia as Substrate in Atrial Fibrillation

Coronary ischemia, even in microvascular circulation specific for NOCAD, may be a significant promotor for AF, the most common sustained cardiac arrhythmia. Coronary ischemia has been identified as a critical substrate in the development and perpetuation of AF, which is why understanding the relationship between coronary ischemia and AF is crucial for developing effective treatment strategies and improving patient outcomes [50].

Coronary ischemia results from an imbalance between myocardial oxygen supply and demand and may be caused by atherosclerosis (in case of obstructive CAD) and other factors such as coronary artery spasm, microvascular dysfunction, and thromboembolism (in case of NOCAD). Regardless of the mechanism, the reduced oxygen supply to the myocardium can cause myocardial injury, inflammation, and fibrosis, creating a substrate conducive to AF. Ischemic episodes cause myocardial cell apoptosis and their replacement with fibrous tissue. This fibrotic tissue disrupts the normal myocardial architecture, creating areas of slow conduction and re-entry circuits, which are critical in the development of AF. Ischemia-induced inflammation further contributes to this process by promoting structural and electrical remodeling of the atria [51].

Moreover, ischemia may also alter the balance of the autonomic nervous system, increasing sympathetic activity and reducing vagal tone. This imbalance can precipitate AF by enhancing automaticity, increasing triggered activity, and promoting re-entry phenomena in the atria. Not least, the complications of coronary ischemia such as heart failure, diastolic dysfunction, and mitral regurgitation will lead to atrial dilatation, promoting AF because, as atrial cardiomyocytes stretch, they are replaced with fibroblasts, which stimulate collagen synthesis, in this way being favored by the genesis of re-entry circuits [52].

Finally, coronary ischemia also plays an important role in the maintenance of AF, not only in its pathogenesis. One study, which involved 700 subjects undergoing a strategy of pulmonary vein isolation for AF, showed that patients with coronary ischemia have a higher recurrence of AF after ablation; moreover, the interventional treatment of coronary lesions can reduce the rate of AF recurrence [53].

Inflammation and oxidative stress are pivotal mechanisms that link coronary ischemia to AF.

### 3.1. Inflammation Due to Coronary Ischemia Leading to AF

When coronary ischemia occurs, it triggers a complex inflammatory response. Reduced blood flow to the myocardium leads to hypoxia, which subsequently causes cellular stress and injury, which will initiate an inflammatory cascade: the first step is the release of cytokines: ischemic myocardial cells release pro-inflammatory cytokines such as interleukin-6 (IL-6), tumor necrosis factor-alpha (TNF-α), and interleukin-1 beta (IL-1β). These cytokines will accelerate fibrosis in atrial cardiomyocytes by modulating matrix metalloproteinase 2 (MMP2) expression and will also recruit inflammatory cells to the site of injury, leading to the second step, the activation of leukocytes: neutrophils, monocytes, and macrophages are among the first responders to ischemic injury, which will release proteolytic enzymes, reactive oxygen species (ROS) and additional cytokines, which further amplify the inflammatory response. Finally, inflammation damages the endothelial cells, leading to increased vascular permeability, reduced nitric oxide availability, and the enhanced adhesion of leukocytes to the endothelium (endothelial dysfunction-the final step) [54,55].

**Figure 3:**
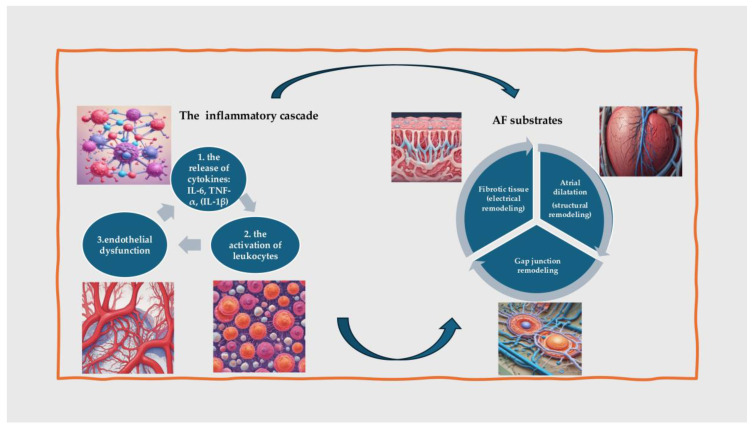
The inflammatory cascade due to coronary ischemia leads to atrial fibrillation. AF: atrial fibrillation; IL: interleukin; TNF: tumor necrosis factor.

The inflammatory cascade will lead to structural and electrical remodeling of the atria, creating a substrate for AF, the main factors being fibrosis, atrial dilation, and gap junction remodeling (Figure 3) [56].

On one hand, prolonged inflammation promotes the activation of fibroblasts and the deposition of extracellular matrix proteins, leading to atrial fibrosis. Fibrotic tissue disrupts normal conduction pathways, creating areas of slow conduction and re-entry circuits that facilitate AF (electrical remodeling). On the other hand, inflammation can lead to atrial dilation, which stretches atrial myocytes and alters their electrophysiological properties. This dilation further predisposes the atria to the development of AF (structural remodeling) [57].

Moreover, inflammatory mediators may also alter the expression and function of connexins, such as connexin 40 and 43, the proteins that form gap junctions between cardiac cells. This disruption of intercellular communication can contribute to the heterogeneous conduction properties seen in AF [58].

### 3.2. Oxidative Stress Due to Coronary Ischemia Leading to Atrial Fibrillation

Reactive oxygen species, which are chemically reactive molecules containing oxygen, play a crucial role in the pathogenesis of AF, particularly in the context of coronary ischemia. During coronary ischemia, the production of ROS increases significantly due to some mechanisms such as mitochondrial dysfunction (ischemia impairs mitochondrial function, leading to an increased production of ROS such as superoxide anions) and activation of NADPH Oxidase and Xanthine Oxidase, two enzymes that generate ROS [59,60].

Reactive oxygen species may contribute to the initiation and perpetuation of AF through several mechanisms: direct myocyte damage, calcium handling abnormalities, ion channel dysfunction, and gap junction dysfunction [61].

Firstly, ROS can directly damage atrial myocytes by oxidizing lipids, proteins, and DNA. ROS-induced cellular damage may lead to apoptosis or necrosis, contributing to atrial fibrosis and inflammation (damaged cells release inflammatory mediators that further propagate inflammation and remodeling in the atria) [62,63].

Calcium ions (Ca^2^⁺) play a crucial role in cardiac myocyte contraction and electrical activity. Secondly, ROS can disrupt normal calcium handling by sarcoplasmic reticulum (SR) dysfunction. They can alter the function of SR Ca^2^⁺-ATPase (SERCA) and ryanodine receptors (RyR), leading to abnormal Ca^2^⁺ release and reuptake; moreover, this dysfunction is the cause of Ca^2^⁺ overload in the cytoplasm. Moreover, the oxidative modification of RyR can cause a “leaky” SR, resulting in spontaneous Ca^2^⁺ release, which can trigger afterdepolarizations and ectopic activity, promoting AF [64].

Then, ROS can also modify the function of various ion channels, which are critical for maintaining normal cardiac electrophysiology, such as:

- sodium channels (Na⁺ Channels): ROS can reduce sodium current (INa) by modifying channel proteins, leading to slowed conduction and increased susceptibility to re-entry circuits [65].
- potassium channels (K⁺ Channels): ROS can affect several potassium channels involved in repolarization, such as IKs, IKr, and Ito. This can prolong or shorten action potential duration, creating a substrate for AF [66].
- calcium channels (Ca^2^⁺ Channels): ROS can increase L-type calcium current (ICa,L), contributing to abnormal calcium influx and triggered activity [67].

Finally, ROS may impair gap junction function by altering the expression and function of connexins (such as connexin 43), leading to impaired intercellular communication, in this way creating a decreased conductance (dysfunctional gap junctions result in heterogeneous conduction and the formation of re-entry circuits, which are key mechanisms in AF) [68].

## 4. Atrial Fibrillation as Substrate for Microvascular Dysfunction

While AF is well recognized for its potential to cause thromboembolic events, its role as a substrate for microvascular dysfunction and coronary ischemia is less understood. Traditionally, the focus has been on the management of stroke risk and rhythm control. However, emerging evidence suggests that AF may also contribute to microvascular dysfunction and coronary ischemia [69].

**Figure 4:**
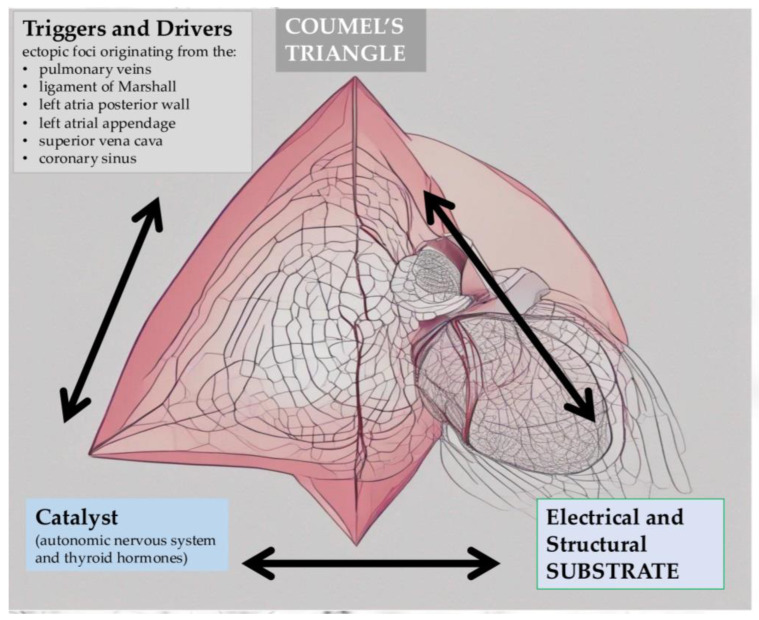
Pathophysiology of atrial fibrillation on the complex relationship between triggers, catalyst and substrate (arrows).

The pathophysiology of AF involves a complex interplay of various factors, being associated with a concept using Coumel’s Triangle that requires a trigger for initiation, a catalyst agent, and a substrate (arrhythmogenic and structural) for the perpetuation and maintenance of the trigger (Figure 4) [51]:

- Triggers and Drivers: AF may be triggered by ectopic beats originating from the pulmonary veins or other locations in the atria, such as the left atria posterior wall, the superior vena cava, the left atrial appendage, the coronary sinus, and the ligament of Marshall. These triggers, combined with areas of slowed conduction and functional re-entry circuits, create a substrate for the initiation and perpetuation of AF. In some cases, rapid firing of ectopic foci or localized reentrant circuits may also drive the arrhythmia [70].
- Catalyst: The catalyst may change refractory periods, in this way increasing autonomic activity, and it is represented by the autonomic nervous system, thyroid hormones, and illicit drugs. Sympathetic activation can increase the likelihood of AF episodes, while parasympathetic stimulation may promote the termination of AF; in this way, imbalances in autonomic tone can influence the susceptibility to AF [71].
- Substrate: The substrate is essential in maintaining the action of trigger and catalyst and it can be structural or electrical. The first one consists of structural modifications in the atria, such as fibrosis (excessive deposition of collagen and other extracellular matrix proteins) and dilation. These changes can disrupt normal electrical conduction pathways in the atrium and create conditions that are conducive to sustaining AF. The second one is created by abnormal electrical activity in the atria, characterized by alterations in ion channel function and intracellular signaling pathways. Modifications in the action potential duration, refractoriness, and conduction velocity can promote the re-entry of electrical impulses and the chaotic electrical activity seen in AF [72].

There are other factors that sustain the activity of the Coumel’s Triangle, such as inflammation and oxidative stress, which may promote atrial structural remodeling, electrical instability, and fibrosis, creating a proarrhythmic environment in the atria and comorbidities (hypertension, heart failure, diabetes, obesity, and sleep apnea) that can contribute to atrial remodeling [61].

Coronary ischemia in AF patients may occur even in the absence of significant epicardial CAD. AF may promote coronary ischemia through a lot of mechanisms such as an increased myocardial oxygen demand (the rapid and irregular ventricular rate during AF increases myocardial oxygen consumption, generating a mismatch between demand and supply of oxygen), reduced myocardial perfusion (AF can lead to reduced diastolic filling time and impaired coronary perfusion, especially during tachycardia) and coronary microembolization (the formation and dislodgement of microthrombi can lead to microvascular obstruction and ischemia) [73].

### 4.1. Microvascular Dysfunction in AF

Despite coronary ischemia, AF may also induce microvascular dysfunction through several mechanisms: endothelial dysfunction, neurohormonal activation, microthrombi formation, and impaired coronary flow reserve [74].

### 4.2. Endothelial Dysfunction Due to Atrial Fibrillation

Endothelial dysfunction, which plays a significant role in the pathophysiology of microvascular dysfunction and coronary ischemia, may be promoted by AF in many ways [75]:

- Systemic inflammation and oxidative stress: On one hand, AF is associated with elevated levels of inflammatory markers such as C-reactive protein (CRP), IL-6, and TNF-α. These cytokines may cause direct damage to endothelial cells, impairing their function. On the other hand, the arrhythmic nature of AF leads to increased production of ROS, which is able to lead to cellular dysfunction and apoptosis [76,77].
- Hemodynamic shear stress: The irregular and rapid heart rate in AF results in abnormal shear stress on the blood vessel walls. This mechanical stress can disrupt the normal function of endothelial cells, reducing their ability to regulate vascular tone and blood flow [78].
- Endothelial nitric oxide synthase (eNOS) dysfunction: In AF, the bioavailability of NO, which is a critical vasodilator produced by endothelial cells, is reduced due to increased oxidative stress and inflammation, leading to impaired vasodilation and endothelial dysfunction. Moreover, under normal conditions, eNOS produces NO; however, in the presence of oxidative stress and reduced tetrahydrobiopterin (BH4), a cofactor for eNOS, the enzyme becomes uncoupled and produces superoxide instead of NO, further exacerbating oxidative stress and endothelial dysfunction [79].

Endothelial dysfunction also contributes to the pro-thrombotic state in AF. The damaged endothelium expresses more adhesion molecules, promoting platelet aggregation and clot formation, which can lead to thromboembolic events. Finally, endothelial dysfunction can exacerbate heart failure by impairing myocardial perfusion and contributing to adverse ventricular remodeling. The reduced NO availability and increased oxidative stress may affect myocardial contractility and promote fibrosis [79,80,81].

### 4.3. Neurohormonal Activation Due to Atrial Fibrillation

Neurohormonal activation plays a crucial role as a catalyst in the pathophysiology of AF and contributes significantly to microvascular dysfunction. This complex interplay involves the activation of the sympathetic nervous system (SNS) and the renin–angiotensin–aldosterone system (RAAS), which collectively exacerbate vascular and cardiac abnormalities [82].

On one hand, AF is associated with heightened sympathetic activity, leading to increased levels of catecholamines (epinephrine and norepinephrine), which bind to adrenergic receptors on endothelial cells and vascular smooth muscle cells, causing vasoconstriction and reduced endothelial-dependent vasodilation. Moreover, catecholamines may also stimulate the production of ROS within endothelial cells, contributing to oxidative stress, which impairs eNOS activity and reduces NO bioavailability. Then, SNS activation promotes the expression of pro-inflammatory cytokines and adhesion molecules on endothelial cells, facilitating leukocyte adhesion and infiltration. This inflammatory response further damages the endothelium and impairs its function [83].

On the other hand, AF stimulates the RAAS, leading to increased production of angiotensin II, which is a potent vasoconstrictor that directly affects endothelial cells by promoting oxidative stress, inflammation, and cellular apoptosis. It also stimulates the production of endothelin-1, another vasoconstrictor that exacerbates endothelial dysfunction. An increased level of aldosterone contributes to endothelial dysfunction by promoting fibrosis, oxidative stress, and inflammation, as it is able to induce the expression of pro-inflammatory cytokines and adhesion molecules, which facilitate leukocyte adhesion and vascular inflammation. In this way, angiotensin II and aldosterone promote vascular remodeling, characterized by increased smooth muscle cell proliferation, fibrosis, and reduced elasticity of blood vessels. This remodeling impairs endothelial function and may contribute to the progression of atherosclerosis. Finally, angiotensin II and aldosterone may also activate NADPH oxidase, an enzyme complex responsible for the production of ROS [84,85].

Not least, chronic RAAS activation leads to sustained vasoconstriction and sodium retention, contributing to the development and maintenance of hypertension, a common risk factor for both AF and coronary ischemia [86].

### 4.4. Microthrombi Formation Due to Atrial Fibrillation

Microthrombi formation is a critical and often underappreciated mechanism by which AF contributes to microvascular dysfunction and coronary ischemia because they are able to obstruct the microvasculature, leading to impaired tissue perfusion and ischemic damage [87].

It is well known that AF, being associated with irregular atrial contraction, is associated with blood stasis, particularly in the left atrial appendage. Then, the irregular ventricular response in AF further contributes to turbulent blood flow, promoting conditions favorable for thrombus formation; however, AF is also associated with endothelial injury, which disrupts the balance between pro-coagulant and anticoagulant factors, favoring thrombus formation. Patients with AF often exhibit elevated levels of coagulation factors such as fibrinogen, factor VIII, and von Willebrand factor. Moreover, AF may lead to increased platelet activation and aggregation, releasing pro-thrombotic substances like thromboxane A2 and adenosine diphosphate, promoting further clot formation [88].

In this way, the microthrombi formed can lodge in the coronary microvasculature, obstructing blood flow and leading to localized ischemia, which is particularly detrimental in the myocardial tissue, that requires high oxygen levels. Recurrent formation and dissolution of microthrombi may also lead to cycles of ischemia and reperfusion injury, causing further endothelial damage and perpetuating a vicious cycle of thrombosis and dysfunction [89,90,91].

The microthrombi formed are responsible for thromboembolic events, not only in the coronary tree but also in other systemic arteries, leading to ischemia in all territories and being one of cause for the cryptogenic stroke [92].

### 4.5. Impaired CFR Due to Atrial Fibrillation

Atrial fibrillation can significantly impact coronary circulation, leading to impaired CFR by several mechanisms. Firstly, the irregular ventricular rate in AF leads to variations in cardiac output and coronary perfusion. The inconsistent filling times and rapid heart rates reduce diastolic filling, a critical period for coronary blood flow, particularly to the left ventricle. Secondly, the loss of atrial systole reduces stroke volume, leading to decreased perfusion pressure in the coronary arteries, which further impairs CFR [93,94].

Then, as discussed previously, AF promotes the formation of microthrombi in the coronary microvasculature, thus limiting the ability of coronary vessels to increase blood flow during periods of increased demand. Moreover, AF-induced neurohormonal activation and inflammation can lead to structural changes in the coronary microvasculature, including increased stiffness and reduced compliance, these changes impair the ability of the vessels to dilate appropriately, reducing CFR [95,96].

Vice versa, impaired myocardial perfusion can contribute to the recurrence and persistence of AF as ischemia-induced atrial remodeling and fibrosis may create a substrate for AF perpetuation, but severe ischemia and impaired CFR can also increase the risk of ventricular arrhythmias, posing a risk for sudden cardiac death [97].

Most patients with AF and impaired CFR will experience symptoms of angina, particularly during periods of increased cardiac demand, such as physical exertion or emotional stress, but some of them can have ischemia without typical anginal symptoms, which can go unnoticed and lead to more severe cardiac events [98].

## 5. NOCAD and AF: Challenges in Clinical Practice

NOCAD and AF represent significant and often overlapping cardiovascular pathologies whose coexistence presents unique challenges in clinical practice, encompassing diagnostic complexities, therapeutic dilemmas, and prognostic uncertainties.

### 5.1. Diagnostic Challenges

The diagnosis of NOCAD in patients with AF is particularly challenging due to the overlapping symptomatology and the limitations of traditional diagnostic modalities. Both conditions can present with angina, dyspnea, and palpitations, making it difficult to distinguish between ischemic symptoms and arrhythmia-related discomfort [99].

While ECG is a cornerstone in the diagnosis of both conditions, its utility is limited in the presence of AF because this arrhythmia can mask ischemic changes such as ST-segment depression or T-wave inversions, complicating the diagnosis of NOCAD. Then, standard coronary angiography may reveal non-obstructive lesions, but it does not provide information on microvascular dysfunction or endothelial dysfunction, which are often seen in NOCAD. Advanced imaging techniques such as IVUS or OCT can be useful but are not routinely performed due to cost and accessibility issues [100,101].

Finally, non-invasive imaging modalities like CCTA and CMR can provide detailed anatomical and functional information, but their interpretation can be complicated by AF-related motion artifacts [102].

### 5.2. Therapeutic Challenges

The treatment of patients with both NOCAD and AF requires a multifaceted approach that addresses both the ischemic and arrhythmic components of their disease. However, the therapeutic strategies for these conditions can sometimes be conflicting.

Regarding antithrombotic therapy, patients with AF are at increased risk of thromboembolic events and usually require anticoagulation. However, those with NOCAD may also require antiplatelet therapy. The concomitant use of anticoagulants and antiplatelets increases the risk of bleeding, necessitating a careful balancing act [103].

The primary anticoagulants used in AF include direct oral anticoagulants (DOACs) such as apixaban, rivaroxaban, dabigatran, and edoxaban, which have taken the place of warfarin. For NOCAD, antiplatelet agents such as aspirin or P2Y12 inhibitors (e.g., clopidogrel, ticagrelor) are commonly prescribed. The challenge lies in determining the necessity and duration of SAPT (single antiplatelet therapy)/DAPT in addition to anticoagulation because the concurrent use of anticoagulants and antiplatelets significantly increases the risk of bleeding; however, the AUGUSTUS trial sustains that, in the case of both AF and coronary ischemia, apixaban and a P2Y12 inhibitor should be used instead of warfarin or dual antiplatelet agents. Moreover, individualized patient assessment using tools like the HAS-BLED score to estimate bleeding risk should be used and balanced against the thrombotic risks. Strategies to mitigate bleeding risk include the use of proton pump inhibitors (PPIs) to prevent gastrointestinal bleeding and close monitoring of coagulation parameters [104,105,106].

Managing AF involves strategies related to rate vs. rhythm control, and both approaches have implications for patients with NOCAD. If it is possible, the conversion to sinus rhythm using antiarrhythmic drugs, electrical cardioversion, or catheter ablation is vital in reducing oxygen consumption, thus preventing the evolution of coronary ischemia. Rhythm control involves the use of antiarrhythmic drugs (AADs) like amiodarone, dofetilide, flecainide, or sotalol. However, the use of flecainide is limited in patients with coronary ischemia (especially post-myocardial infarction), as it may increase mortality in this case; however, a study with only 78 patients claimed that treatment with flecainide appears not to increase mortality in patients with AF, preserved left ventricle function, and occult CAD indicated by PET Stress Testing with Coronary Flow Capacity. Moreover, another study sustains the effectiveness and safety of dofetilide in patients with AF and heart failure or CAD [107,108].

Beyond the antiarrhythmic drugs, mineralocorticoid receptor antagonists and sodium glucose cotransporter 2 inhibitors have shown promising effects in the management of new-onset or recurrent atrial fibrillation. Mineralocorticoid receptor antagonists such as spironolactone and eplerenone have been found to exert antifibrotic and anti-inflammatory effects on the atrial myocardium, potentially reducing the structural remodeling that predisposes to atrial fibrillation. These drugs also exhibit antiarrhythmic properties by modulating ion channels and reducing oxidative stress. On the other hand, sodium glucose cotransporter 2 inhibitors like empagliflozin and canagliflozin have demonstrated beneficial cardiovascular effects beyond glycemic control in patients with diabetes. They have been associated with reduced incidence of atrial fibrillation by promoting diuresis, natriuresis, and weight loss, which, collectively, alleviate cardiac strain and reduce the risk of atrial fibrillation. Furthermore, these agents exhibit favorable effects on ventricular function and systemic hemodynamics, contributing to their potential anti-arrhythmic effects in atrial fibrillation. Overall, the combination of mineralocorticoid receptor antagonists and sodium glucose cotransporter 2 inhibitors presents a novel therapeutic strategy for managing atrial fibrillation by targeting both the underlying pathophysiological mechanisms and associated comorbidities [109,110].

If AF is declared permanent, rate control is typically achieved using beta-blockers, calcium channel blockers, or digoxin. Beta-blockers are also the first-line treatments included in antianginal medications while calcium channel blockers are the first option for vasospastic angina. Digoxin, while effective in controlling heart rate, has a narrow therapeutic index and potential proarrhythmic effects [104].

Catheter ablation is an option for rhythm control in patients with symptomatic AF refractory to medical therapy. By restoring normal heart rhythm through ablation, the risk of microvascular dysfunction, endothelial instability, and subsequent ischemic events can be mitigated. Consequently, AF ablation may not only improve symptomatic relief for patients but also enhance overall cardiovascular outcomes by addressing the underlying electrophysiological disturbances that contribute to ANOCA, INOCA, and MINOCA. This underscores the importance of considering AF ablation in the broader context of cardiovascular disease management, particularly for patients exhibiting these complex, non-obstructive coronary conditions. However, the procedure itself carries risks, and its impact on coronary microvascular function in NOCAD patients is not fully understood. Moreover, the recurrence of AF post-ablation remains a concern, necessitating ongoing monitoring and potential repeat procedures [111].

### 5.3. Prognostic Challenges

Understanding the prognosis in patients with coexisting NOCAD and AF is complex, as each condition independently influences cardiovascular outcomes. All types of AF (paroxysmal, persistent, or permanent) should be closely monitored for different subtypes of NOCAD. Regarding vice-versa, especially MINOCA patients should be monitored for AF development.

Risk stratification tools for AF and NOCAD may not fully capture the interplay between these conditions. For example, in the CHA2DS2-VASc score, it is included obstructive CAD but not NOCAD. There are studies that claim that the CHA2DS2-VASc score may be a useful predictor for selecting patients to undergo coronary angiographic exploration for the diagnosis of obstructive CAD or that it may be an independent predictor for SYNTAX score, in patients with non-ST-segment elevation acute myocardial infarction and without AF, but no correlations with NOCAD have been studied yet [112,113].

Comprehensive risk models that integrate both ischemic and arrhythmic risks are needed. Then, studies suggest that patients with NOCAD and AF have a higher incidence of adverse cardiovascular events compared to those with either condition alone. The mechanisms underlying this increased risk are multifactorial, involving endothelial dysfunction, chronic inflammation, and neurohormonal activation [114].

Finally, both NOCAD and AF significantly impact patients’ quality of life. The recurrent symptoms, frequent hospitalizations, and the psychological burden of managing multiple medications can lead to reduced adherence and poorer outcomes [115].

## 6. Key Points

The dual relationship between NOCAD and AF is a significant area of interest due to its complex clinical implications. The key points of this relationship include the increased propensity for patients with NOCAD to develop AF due to shared risk factors such as hypertension, diabetes, and obesity. Moreover, NOCAD can exacerbate the symptoms and progression of AF, leading to a higher incidence of adverse cardiovascular events. The presence of AF in NOCAD patients also complicates the management strategies, necessitating a comprehensive approach that addresses both rhythm control and the underlying coronary microvascular dysfunction. Understanding this dual relationship is crucial for optimizing diagnostic, therapeutic, and preventive measures to improve patient outcomes. In Table 3, we summarized some studies that claim the dual relationship between these pathologies and their challenges in clinical practice.

## 7. Conclusions

NOCAD and AF seem to be a complex interplay with one begets another. The coexistence of NOCAD and AF presents a myriad of challenges in clinical practice. Diagnostic accuracy is hindered by overlapping symptoms and limitations of current imaging modalities. Therapeutic strategies must balance the risks of bleeding and thromboembolism, while also managing ischemic and arrhythmic components effectively. Prognostic evaluation is complicated by the intricate interplay between the two conditions, necessitating more sophisticated risk models. Addressing these challenges requires a multidisciplinary approach, integrating cardiology, electrophysiology, and patient-centered care to optimize outcomes for this complex patient population. Future research should focus on developing targeted therapies and refining diagnostic tools for better management.

## 8. Future Perspectives

The relationship between NOCAD and AF presents a promising yet underexplored area for future cardiovascular research. Despite advancements in understanding coronary artery diseases and arrhythmias independently, the interplay between NOCAD and AF remains insufficiently elucidated. Future perspectives should focus on large-scale, longitudinal studies to establish causal relationships and underlying mechanisms linking these conditions. Research gaps include the need for more precise biomarkers and imaging techniques to detect subclinical inflammation and fibrosis that might bridge NOCAD and AF. Additionally, exploring genetic predispositions and the role of microvascular dysfunction could provide deeper insights. Addressing these gaps can potentially revolutionize preventive strategies and therapeutic interventions, ultimately improving patient outcomes in those concurrently suffering from NOCAD and AF.

## Figures and Tables

**Table 1:** Diagnostic tools used to identify ANOCA and INOCA. ANOCA: Angina with Non-Obstructive Coronary Arteries; CAD: coronary artery disease; FFR: Fractional Flow Reserve; HMR: hyperemic myocardial velocity resistance; IFR: Instantaneous Wave-Free Ratio; IMR: index of microvascular resistance; INOCA: Ischemia with Non-Obstructive Coronary Arteries; MINOCA: Myocardial Infarction with Non-Obstructive Coronary Arteries; CFR: coronary flow reserve.

Diagnostic Tool	Role of the Diagnostic Tool
Cardiac biomarkers (especially high-sensitive troponin) [23]	May differentiate ANOCA and INOCA from MINOCA by showing the status of myocardial injury
Non-invasive tests to detect ischemia: exercise tolerance test, transthoracic doppler echocardiography, myocardial contrast echocardiography, myocardial perfusion imaging, positron emission tomography, stress echocardiography, and cardiac MRI [24]	May differentiate ANOCA (without signs of ischemia) from INOCA (the signs of ischemia are present)
Coronary angiography [25]	Gold standard to exclude obstructive CAD
Invasive tests during coronary angiography: vasoreactivity test using intracoronary acetylcholine, FFR, IFR, CFR, IMR, HMR [26]	May differentiate the type of NOCAD regarding its physiopathology

**Table 2:** Types of SCAD based on angiography.

Type of SCAD	Description
Type 1 (contrast staining of false lumen)	It has contrast stains in the arterial wall with multiple radiolucent lumens with or without slow contrast clearing.
Type 2 (long diffuse and smooth narrowing)	It shows diffuse, smooth, usually, 20–30 mm narrowing with varying severity.
Type 3 (focal/tubular stenosis)	It shows focal or tubular stenosis that mimics atherosclerosis.
Type 4 (occlusion of the vessel)	There is no antegrade flux distal to the lesion.

**Table 3:** The main studies which assessed the mechanisms between NOCAD and AF. CFR: coronary flow reserve, CAD: coronary artery disease, CCTA: computed coronary angiography, PET-CFC: coronary flow capacity on positron emission tomography, AADs: anti-arrhythmic drugs, CAG: coronary angiography.

Trial		Mechanism	Methods	Conclusions
1. Nso N et al., 2020 [116]	Coronary ischemia as substrate in AF	Inflammation	An in-depth review of the available literature criteria number	The inflammatory cascade induces fibrotic changes in the myocardium, an arrhythmogenic process that may promote AF
2. Oktay V et al., 2014 [117]		Oxidative stress	Single-center study with 118 patients	The role of oxidative stress related to ischemia-reperfusion damage may be a key to the pathogenesis of AF
3. Corban MT et al., 2021 [69]	AF as a substrate for microvascular dysfunction	Endothelial Dysfunction	An in-depth review of the available literature criteria number	Experimental and clinical studies have shown that AF is associated with systemic vascular and atrial endothelial dysfunction
4. Charitakis et al., 2016 [118]		Neurohormonal Activation	Randomized Controlled Study including 45 patients	AF is a strong stimulus that causes an immediate activation of different biomarkers and has an immediate effect on hemodynamics leading to neurohormonal activation
5. Violi F et al., 2014 [119]		Microthrombi Formation	Review	AF is characterized by a constellation of atherosclerotic risk factors, including hypertension, dyslipidemia, and diabetes, which may predispose to serious clinical complications of atherosclerosis
6. Ozcan C et al., 2021 [120]		Impaired CFR	Cohort of 80 patients	Patients with AF were more likely to have coronary microvascular dysfunction
7. Rottländer D et al., 2021 [102]	Challenges in clinical practice	Diagnostic challenge	A 5-year single-center retrospective analysis with 566 patients	Patients with paroxysmal or first-diagnosed AF are at risk for CAD, while CCTA is a feasible diagnostic tool for CAD
8. Pantlin PG et al., 2020 [108]		Therapeutic challenge	Pilot study with 78 patients	In a limited population of AF patients with preserved left ventricle function and PET-CFC indicating occult CAD, treatment with 1C AADs appears not to increase mortality
9. Wojszel et al., 2022 [113]		Prognostic challenge	A cross-sectional study with 452 patients	Several older AF patients who are advised to undergo elective CAG have nonobstructive CAD

## Data Availability

The data are contained within this article.
